# Cortical reorganisation of cerebral networks after childhood stroke: impact on outcome

**DOI:** 10.1186/s12883-015-0309-1

**Published:** 2015-06-10

**Authors:** Salome Kornfeld, Juan Antonio Delgado Rodríguez, Regula Everts, Alain Kaelin-Lang, Roland Wiest, Christian Weisstanner, Pasquale Mordasini, Maja Steinlin, Sebastian Grunt

**Affiliations:** Division of Neuropaediatrics, Development and Rehabilitation, Children’s University Hospital, Inselspital, Bern, Switzerland; Center for Cognition, Learning and Memory, University of Bern, Bern, Switzerland; Neurocenter of Southern Switzerland, Lugano, Switzerland; Department of Diagnostic and Interventional Neuroradiology, University Hospital, Inselspital, Bern, Switzerland; Graduate School for Health Sciences, University of Bern, Bern, Switzerland

**Keywords:** Cortical reorganisation, Functional networks, Neuroplasticity, Paediatric arterial ischaemic stroke, Resting state functional magnetic resonance imaging, Transcranial magnetic stimulation

## Abstract

**Background:**

Recovery after arterial ischaemic stroke is known to largely depend on the plastic properties of the brain. The present study examines changes in the network topography of the developing brain after stroke. Effects of brain damage are best assessed by examining entire networks rather than single sites of structural lesions. Relating these changes to post-stroke neuropsychological variables and motor abilities will improve understanding of functional plasticity after stroke. Inclusion of healthy controls will provide additional insight into children’s normal brain development. Resting state functional magnetic resonance imaging is a valid approach to topographically investigate the reorganisation of functional networks after a brain lesion. Transcranial magnetic stimulation provides complementary output information.

This study will investigate functional reorganisation after paediatric arterial ischaemic stroke by means of resting state functional magnetic resonance imaging and transcranial magnetic stimulation in a cross-sectional plus longitudinal study design. The general aim of this study is to better understand neuroplasticity of the developing brain after stroke in order to develop more efficacious therapy and to improve the post-stroke functional outcome.

**Methods:**

The cross-sectional part of the study will investigate the functional cerebral networks of 35 children with chronic arterial ischaemic stroke (time of the lesion >2 years). In the longitudinal part, 15 children with acute arterial ischaemic stroke (shortly after the acute phase of the stroke) will be included and investigations will be performed 3 times within the subsequent 9 months. We will also recruit 50 healthy controls, matched for age and sex. The neuroimaging and neurophysiological data will be correlated with neuropsychological and neurological variables.

**Discussion:**

This study is the first to combine resting state functional magnetic resonance imaging and transcranial magnetic stimulation in a paediatric population diagnosed with arterial ischaemic stroke. Thus, this study has the potential to uniquely contribute to the understanding of neuronal plasticity in the brains of healthy children and those with acute or chronic brain injury. It is expected that the results will lead to the development of optimal interventions after arterial ischaemic stroke.

## Background

Paediatric arterial ischaemic stroke (AIS) has an incidence of at least 2.1:100,000 children per year [1]. It is a rare but devastating event that impacts the lives of affected children, their families, and individuals in their social environment [2]. Paediatric AIS has an estimated mortality of up to 20% [3] and is a considerable cause of childhood morbidity [4,5]. Two-thirds of patients who experience AIS suffer from lifelong cognitive or neurological handicaps [2,6]. It is important to have solid models to enable prediction of recovery after paediatric AIS, not only to provide useful information for parents and children, but also to develop and choose potentially beneficial interventions.

Recovery after AIS largely depends on plastic properties of the brain. The term neuroplasticity refers to the ability of the central nervous system to adapt to changes in the external and internal milieu [7] and is associated with structural and functional modifications in the brain which can be detected via neuroimaging and neurophysiological methods. To improve the functional outcome of children after a brain injury, a deeper understanding of processes driving neuroplastic changes is crucial [8].

There is an ongoing debate on whether or not young age is advantageous with regard to brain plasticity. “Young age plasticity privilege” or the “Kennard-effect” derive from studies describing superior recovery of cognitive and motor skills after brain lesions in infant animals and humans compared to adults [9-11], attributed to superior plasticity in the immature brain. Other studies support an “early vulnerability” concept, in which young brains are especially vulnerable to strokes [12-15]. A third, more recent perspective merges both extremes and considers outcome after brain lesions to be influenced by different factors such as age at injury and sociodemographic factors, oscillating on a “recovery continuum” between plasticity and vulnerability [16].

Recent developments suggest that functional recovery after brain injury is strongly dependent on changes in widely distributed neuronal networks controlling brain functions [17]. This network perspective suggests that effects of a brain injury are best assessed by examining entire networks rather than single sites of structural damages or adjacent regions [18]. Cerebral network maturation is a long lasting process [19], and functional connections between different regions change with age [20]. During childhood, functional connections to distant regions become stronger with advancing maturation [21]. Furthermore, certain networks, such as the default mode network [20], are only slightly functionally connected in childhood but increase in connection strength over time until they are fully developed by adulthood [20]. Thus, an early childhood stroke that affects immature connections might have a wider impact on functional reorganisation than a stroke affecting more mature networks.

For visualisation of paediatric brain networks and their changes during development or after injury, resting state functional magnetic resonance imaging (rsfMRI) is one of the most appropriate techniques. Resting state fMRI measures the temporal correlation of the blood oxygen level dependent (BOLD) signal between different brain regions at rest. It is particularly suited to examine lesion effects on proximal and distal brain areas by displaying different networks across the whole brain [22] and also reduces scanning time if multiple networks are being investigated [23]. Therefore, it provides information about lesion effects in a short time period (<10 minutes) and requires no active participation, both of which are useful for research with handicapped and/or young children [22,23].

Findings of rsfMRI studies in adult patients after AIS suggest that the functional connectivity between the ipsilesional and the contralesional hemisphere in a resting state situation is predictive for motor and cognitive outcome [24-26]. For example, He et al. (2007) showed that functional connectivity between the left and right hemispheric posterior intraparietal sulcus was reduced in acute stroke patients but fully recovered at the chronic stage. This corresponded with improved behavioural performance from acute to chronic stage [24]. Concerning motor recovery, rsfMRI data revealed greater functional connectivity with ipsilesional structures, and decreased functional connectivity with the contralesional motor cortex [25]. Motor recovery 6 months post-stroke was positively correlated with functional connectivity of the ipsilesional motor cortex with the contralesional thalamus, supplementary motor area, and middle frontal gyrus at time of acute stroke [25].

To our knowledge, only Dinomais et al. (2012) have published results of rsfMRI in paediatric AIS patients [27]. The authors applied rsfMRI to a relatively small sample of children after neonatal stroke and analysed the relationship between functional connectivity and sensory impairment. Children who had lesions in the middle cerebral artery displayed significantly less functional connectivity in the somatosensory cortex than children who had periventricular lesions. However, this difference disappeared after correction for the loss of cortical grey-matter volume. The authors concluded that rsfMRI is a valid approach to investigate functional networks after brain lesions in children, noting especially the advantage of independence from compliance and level of performance [27].

Besides neuroimaging techniques, neurophysiological technologies such as transcranial magnetic stimulation (TMS) have contributed to the understanding of functional reorganisation after AIS [28-34]. TMS is a safe, non-invasive technique that uses direct stimulation of the brain for diagnostic and therapeutic purposes in neurologically impaired adults [35] and children [36]. Among other applications, TMS has been used to examine the existence of descending ipsilesional and/or contralesional corticospinal projections in hemiparetic patients [37] and also to analyse the central motor facilitatory and inhibitory processes, which allows study of interhemispheric inhibition (IHI), a physiological process in which one hemisphere inhibits its contralateral homologous counterpart [28,38]. After AIS, adult patients show increased IHI [28]. Despite the small number of studies, some evidence supports the presence of this phenomenon in children after AIS [34]. Enhanced IHI after AIS correlates with adverse motor outcome [28,38-40].

Whereas rsfMRI provides a detailed topographical analysis about the location and extent of cerebral networks, it does not provide information about the activity of these networks. In contrast, no particular information about spatial properties of neural networks is available from TMS, but it is especially suited to assess activational remapping of the sensorimotor cortex after a lesion. In order to gather information about both the extent and the activity of cerebral networks, parallel use of rsfMRI and TMS are optimal complementary measures.

To date, neither a cross-sectional nor a longitudinal study has assessed the process of cortical reorganisation using a combination of TMS and neuroimaging technologies in children recovering from AIS. Therefore, the aim of the proposed study is to investigate post-stroke plasticity characteristics over time, with comparison to healthy controls, using an approach that covers both mapping and outcome of network changes. We generally assume that changes in networks over time compared to healthy controls will be correlated with changes in neuropsychological domains and in motor functions. Furthermore, the findings will provide new insights into paediatric brain development. The study could substantially contribute to the broad discussion of “young age plasticity”.

## Methods

### Study design

#### Cross-sectional evaluation

In the cross-sectional part, only patients who had AIS at least two years earlier will be considered for inclusion. Patients will be identified by the Swiss Neuropaediatric Stroke Registry (SNPSR). The SNPSR is a multicentre prospective population-based registry in Switzerland that includes children who experienced AIS at age ≤16 y. The SNPSR was approved by the Cantonal Ethic Committee of Bern, Aarau, Basel and Lucerne as well as by the Swiss Federal Ministry of Health. Parents of eligible patients will be directly contacted. Resting state fMRI, TMS, neuropsychological testing, hand function assessment and disease specific outcome will be assessed once. Specific outcome will each be assessed at a separate appointment. The interval between appointments must not exceed one week. The aim of the cross-sectional part is to perform a cross-comparison of patients after stroke with age- and gender-matched healthy controls in order to detect possible long-term abnormalities in networks and functional domains after childhood AIS.

#### Longitudinal evaluation

In the longitudinal part, only patients who had AIS within the previous two years (i.e. acute phase) will be considered for inclusion. Parents of affected children will be directly contacted. At the time of the acute stroke event, MRI or computer tomography (CT) are performed for diagnostic purposes by the corresponding local centres according to local guidelines with suggestions from the SNPSR. One month and 9 months after the acute stroke onset a neurological exam, paediatric stroke outcome measurement (PSOM), neuropsychological testing, hand function assessment, rsfMRI and TMS will be performed. Intervals between different assessments must not exceed one week. Additionally, rsfMRI will be performed at three months post-AIS. The aim of the longitudinal part is to investigate post-stroke changes in the network topography and functional domains over time, and to compare those changes to age- and gender-matched healthy controls.

### Participants

Written informed consent for participation in the study was obtained from patients and controls themselves if they were older than 18 years. For children younger than 18 years, a parent or guardian gave the written informed consent.

#### Patients

Patients diagnosed with AIS in Switzerland (confirmed by MRI and/or CT) and aged 5 to ≤16 years at the time of AIS will be eligible for inclusion in this study. Exclusion criteria are ferrous implants, active epilepsy, claustrophobia and behavioural problems that make an MRI or TMS investigation impossible. According to previous data of the SNPSR [1,41,42] we expect an acute AIS sample of approximately 20 children within the study period of 3 years. Considering a potential drop-out rate of 20%, we aim to recruit 15 children with acute AIS for the longitudinal observational evaluation and 35 children with chronic AIS for the cross sectional evaluation.

#### Controls

Age- and gender-matched healthy controls will be recruited at schools and from the personal contacts of patients and study employees. Fifty healthy children will be included in the control group.

### Data collection

For each participating child, age, gender, symptoms at stroke manifestation, the Paediatric NIH Stroke Scale (PedNIHSS) [43,44] at manifestation, comorbidities, and information about medication and therapies will be recorded. Outcome measures will be assessed one month and 9 months post-stroke for the longitudinal part, and two years post-stroke for the cross-sectional part.

#### Neuropsychology

All children will undergo an assessment of general intelligence (short form of the Wechsler Intelligence Scale for Children, WISC- IV) [45,46], visuo-spatial perception and visuo-constructive abilities (Developmental Test of Visual-Motor Integration, Beery VMI) [47], attention (Test of Attentional Performance, TAP) [48], verbal and visual memory (Verbal learning and memory test, VLMT [49]; Memory and Learning Test, BASIC-MLT [50]) and executive functions (Animal Naming Fluency Test [51], 5 Point Fluency Test [51], Trail Making Test and word production task of the Delis-Kaplan Executive Function System D-KEFS [52]).

#### Motor abilities

##### Disease specific outcome measure: Paediatric Stroke Outcome Measure (PSOM)

The PSOM assesses neurological deficits and function after childhood AIS, for patient ages ranging from newborn up to adult, on 5 different subscales (right and left sensorimotor, language production, language comprehension, and cognitive/behaviour) [53].

##### Assessment of hand function: Dynamometer, Melbourne Assessment of Unilateral Upper Limb Function and ABILHAND-Kids

Maximal hand strength is assessed by measuring the palmar grasp strength and thumb-forefinger pinch strength with a dynamometer. The maximal effort value will be included for subsequent analyses. The quality of unilateral upper limb movement is examined by means of the Melbourne Assessment of Unilateral Upper Limb Function (MUUL) [54,55]. Manual abilities in daily life are assessed by means of the ABILHAND-Kids in which parents rate their child’s performance on bimanual activities [56].

#### MRI

All MRI images will be acquired using a 3 T Magnetom Verio Siemens scanner (Siemens, Erlangen, Germany). High-resolution T1-weighted MR structural images will be recorded using a magnetisation prepared rapid gradient-echo 3D sequence (repetition time = 2530 ms, inversion time = 1100 ms, echo time = 2.92 ms, 160 sagittal slices, field of view 256 × 256 mm^2^, matrix size 256 × 256 mm^2^), resulting in an iso-voxel resolution of 1 mm^3^ and the use of generalised autocalibrating partially parallel acquisition parallel imaging with an acceleration factor of 2 (acquisition time = 5.05 min) [57]. To minimize head motion, a head support system consisting of two pillows positioned on each side of the head will be used. Earplugs will reduce the scanner noise. Functional imaging will be performed using a multiband echo planar imaging sequence (repetition time = 300 ms, echo time = 30 ms, Distractor factor 0%, Phase oversampling 0%, Multi-band acceleration factor 8, field of view read = 230 mm, field of view phase 100%, pixel size = 3.6 mm × 3.6 mm, 32 axial slices covering the whole brain, slice thickness = 3.6 mm).

#### Transcranial magnetic stimulation

##### Stimulation and recording

Motor evoked potentials (MEPs) will be recorded simultaneously from both forearms using surface electromyography (EMG) electrodes attached over the participant’s Musculus abductor pollicis longus. EMG activity of the Musculus abductor pollicis longus will be monitored to ensure complete relaxation. MEPs will be excluded from the analysis according to a standard protocol [58]. Both hemispheres will be searched for stimulation points eliciting contra- or ipsilateral MEPs. Single, monophasic TMS pulses will be delivered to the motor cortex through a 90 mm round coil connected to a Magstim 200 (the Magstim Company Limited, Whitland, UK). The coil will be positioned such that the cortex areal 5 cm lateral and 1 cm anterior of the vertex is stimulated [59]. The ‘hot spot’ (position eliciting a reproducible muscle response with lowest stimulation intensity) and its resting motor thresholds (RMT; minimum stimulation intensity producing at least five MEPs exceeding 50 μV in ten trials at rest [60]) will be assessed. Latencies will be measured from a superposition of at least three consecutive traces from consecutive stimulations over 110% of RMT.

Participants will be assessed with regard to their cortico motoneuronal organisation (ipsilateral or contralateral descending cortico-spinal motor projections) based on the procedure described by Staudt et al. 2002 [37]. Cortical excitability will be assessed as previously described in a similar population [34]. Suprathreshold intensities of 120%, 130% and 140% of the RMT will be randomly administered (10 stimuli per level, 30 stimuli per side) and stimulus–response curves will be constructed for the stroke and the non-stroke sides. To assess IHI, paired-pulsed stimulation will be used [34,61]. For this purpose, two stimuli will be administered with stimulus intervals of 10 and 40 ms on both hemispheres. A suprathreshold conditioning stimulus (CS) will be administered to the contralateral hemisphere prior to a suprathreshold test stimulus on the stroke side. Interhemispheric inhibition will be expressed by two different components according to two different stimulus intervals (10 ms for short interhemispheric inhibition and 40 ms for long interhemispheric inhibition) [62]. Paired-pulsed monophasic TMS pulses will be delivered to the motor cortex through two figure 8-shaped coils (diameter 5 cm, maximal field strength 2.89 T) connected to a Magstim 200 (Magstim Company Limited, Whitland, UK). Both hemispheres will be stimulated simultaneously and we will assess both directions (stroke to non-stroke and non-stroke to stroke).

### Data analysis

Functional MR-image processing will be performed within Statistical Parametric Mapping (SPM8; http://www.fil.ion.ucl.ac.uk) and will include 3-D motion detection and correction using Levenberg-Marquarts’s least square fit for six spatial parameters, slice scan time correction through Sinc-interpolation. Co-registration of 2-D functional and 3-D structural measurements will be performed and normalisation of data will lead to images in standard definition Montreal Neurological Institute space. All fMRI time-series will be further analysed with CONN fMRI connectivity toolbox 14.n [63] and within the framework of Independent Component Analysis (ICA) using the Group ICA Toolbox (GIFT software) [64] in order to compute the feature of the resting state network and the functional connectivity network. The whole chain of ICA processing will include two main steps: 1) reduction of the dimensionality of data using principal component analysis (PCA), which will also include a reduction of dimensionality at subject level, and 2) concatenation of the reduced data from all subjects and all sessions. The ICA algorithm will consider a total of 20 independent components. We will compute subject maps and identify temporally coherent networks by estimating maximally independent spatial sources. Finally, we will use a back-reconstruction method based on PCA compression and projection to estimate subject-specific subject maps and temporally coherent networks for rest separately for all sessions [65]. From the 20 independent components we will choose a subset of components using a regression analysis that will include rest stimulus condition and the TMS condition. Finally, a subsequent t-test for the beta values corresponding to the rest and TMS condition will be computed [66].

TMS patients will be classified into two patterns of neuronal organisation according to their representation of descending cortico-spinal motor projections (ipsi- or contralateral). Cortical excitability will be expressed separately for the stroke and the non-stroke sides by the RMT and by stimulus response curves, and IHI will be expressed for the stroke to non-stroke and the non-stroke to stroke direction separately, and illustrated by the short interhemispheric inhibition and the long interhemispheric inhibition separately.

In the cross sectional evaluation, neuroimaging, TMS and functional data will be compared between children diagnosed with AIS versus healthy controls. In the longitudinal evaluation, group comparisons will be performed separately after 1, 3 and 9 months. Furthermore, correlation analyses will be performed to compare data on neuroimaging, TMS, and functional assessments. Statistical calculations will be performed with parametric and non-parametric analyses according to the type of data distribution.

## Discussion

Understanding more about functional reorganisation and recovery after stroke might enable better prediction and prevention of post-stroke deficits. Because AIS produces mostly focal, unilateral lesions, it can be used to investigate reorganizational patterns and thus to display how cerebral reorganisation and functional improvement are interrelated. The resulting information could also provide insight into unknown dysfunctional plastic mechanisms, as plastic changes may be maladaptive and do not necessarily lead to functional improvements [11]. Through the additional knowledge about the reorganizational capabilities of a child’s brain, the present study will improve treatment of children with stroke, not only by better adapting specialised education and counselling for patients and parents, but also by providing new insights into specific plasticity-related characteristics, which could help to improve individualized therapeutic rehabilitation procedures.

The human brain has remarkable plasticity throughout the life span. Structural and functional flexibility enables the developing brain to counteract adverse events such as stroke. Compared with adults, who often show long lasting cognitive and motor impairment, children with AIS often show surprisingly good functional outcomes. For these reasons children present a unique study population to learn more about mechanisms of neuroplasticity, particularly because the functional specificity of maturing brains is incomplete [16]. This study has the potential to answer the decades-old question of whether young brains recover and adapt more quickly after injury as compared to older brains.

A major strength of this study is the combination of rsfMRI and TMS. On the one hand, rsfMRI enables visualisation of different neuronal brain networks in the form of functional network maps for injured and healthy children. Subsequent comparisons might reveal direct consequences of stroke lesions on the connectivity between certain brain regions. These visual findings must be correlated with neuropsychological and neurological features, which allow further insight into how network disruptions impact functional outcome. On the other hand, TMS can detect possible imbalances in the interhemispheric motor system after a brain lesion, and the resulting activity can also be correlated with network changes, thereby providing information about the motor outcome after certain network disruptions. Taken together, this study offers a differentiated, non-invasive approach to identify mechanisms of functional recovery in a child’s brain over time.

Research in patients with stroke is not only relevant to unravelling the neuroplastic processes of recovery, but may also provide information about general developmental properties of the normal human brain. This study may also expand our knowledge of the development of neuropsychological and motor functions in the healthy brain. Thus, data regarding functional patterns in the healthy developing brain will be an added benefit of the present study, allowing for definition of normative standards and an improved identification of pathological patterns.

In conclusion, the present study allows the collection of data on a rare but serious clinical condition. It therefore contributes to the overall understanding of the maturing brain, of how it deals with a focal injury, of the role and functioning of underlying reorganisation processes, and eventually of the connection between these processes and the functional outcome.

## Abbreviations

AIS: Arterial ischaemic stroke
BOLD: Blood oxygen level dependent
CS: Conditioning stimulus
CT: Computer tomography
EMG: Electromyography
fMRI: Functional magnetic resonance imaging
ICA: Independent component analysis
IHI: Interhemispheric inhibition
MEPs: Motor evoked potentials
PCA: Principal component analysis
PSOM: Paediatric Stroke Outcome Measure
RMT: Resting motor thresholds
rsfMRI: Resting state fMRI
SNPSR: Swiss Neuropaediatric Stroke Registry
TMS: Transcranial magnetic stimulation
